# Supplementary material for: Correlations between Achilles tendon moment arm and plantarflexor muscle architecture variables
Source: PLoS One. 2024 Aug 29;19(8):e0309406. doi: 10.1371/journal.pone.0309406 (PMC11361577; doi:10.1371/journal.pone.0309406)
Supplement: S1 Appendix — (DOCX) [file pone.0309406.s001.docx]

**S1 Appendix. Accuracy and reliability of three-dimensional ultrasound volume estimations.**

The accuracy and reliability of the ultrasound-based volume measurements were assessed using phantoms that were water-filled condoms. The phantoms were imaged using the three-dimensional ultrasound method described in the main text of the paper, in a manner similar to that used Barber et al. [1], with either single sweep or a double sweep. Each of the phantoms were weighed before and after being filled with water. The net weight of the water was used to compute the volume. The 3DUS method was completed one time using a single and double sweep on 14 and 18 water-filled condom phantoms (S1 Table), respectively, and five times on three incrementally sized (small, medium, and large) phantoms (S2 Table).

**S1 Table. Summary of accuracy and reliability of Single and Double Sweep 3DUS volume measurements.**

| **Number of Sweeps** | **Measured Volume**  **(**$\mathbf{c}\mathbf{m}^{\mathbf{3}}$**)** | **Estimated Volume**  **(**$\mathbf{c}\mathbf{m}^{\mathbf{3}}$**)** | **Difference (**$\mathbf{c}\mathbf{m}^{\mathbf{3}}$**)** | **Percent Error (%)** |
| --- | --- | --- | --- | --- |
| **Single (14x)** | 264.07 ± 48.8 | 263.83 ± 50.1 | -0.24 ± 3.3 | -0.19 ± 0.0 |
| **Double (18x)** | 274.20 ± 49.4 | 269.1 ± 51.3 | -5.14 ± 8.8 | -1.86 ± 0.0 |

**S2 Table. Summary of accuracy and reliability of Double Sweep 3DUS volume measurements.**

| **Phantom Size** | **Measured Volume (**$\mathbf{c}\mathbf{m}^{\mathbf{3}}$**)** | **Estimated Volume (**$\mathbf{c}\mathbf{m}^{\mathbf{3}}$**)** | **Difference (**$\mathbf{c}\mathbf{m}^{\mathbf{3}}$**)** | **Percent Error**  **(%)** | **Coefficient of Variation (%)** |
| --- | --- | --- | --- | --- | --- |
| **Small** | 193.3 | 180.9 ± 0.9 | -12.4 ± 0.9 | -6.4 ± 0.4 | 0.5 |
| **Medium** | 273.5 | 269.9 ± 0.4 | -3.6 ± 0.4 | -1.3 ± 0.1 | 0.1 |
| **Large** | 330.9 | 325.5 ± 1.1 | -5.4 ± 1.1 | -1.6 ± 0.3 | 0.3 |

For the 14 phantom volumes estimated with a single sweep, the average volume was 264.07 ± 48.8 $\mathrm{cm}^{3}$ and percent errors were -0.19 ± 0.0%, and the average volume was 274.20 ± 49.4 $\mathrm{cm}^{3}$ and percent errors were -1.86 ± 0.0% for the 18 phantoms scanned with a double sweep (S1 Table). The coefficient of variation for five 3DUS measurements of the three phantom sizes, small (193.3 $cm^{3}$), medium (273.5 $cm^{3}$), and large (330.9 $cm^{3}$) were 0.5, 0.1, and 0.3%, respectively (S2 Table).

## References

1. Barber L, Barrett R, Lichtwark G. Validation of a freehand 3D ultrasound system for morphological measures of the medial gastrocnemius muscle. J Biomech. 2009;42(9):1313-9.
