# Supplementary material for: Correlations between Achilles tendon moment arm and plantarflexor muscle architecture variables
Source: PLoS One. 2024 Aug 29;19(8):e0309406. doi: 10.1371/journal.pone.0309406 (PMC11361577; doi:10.1371/journal.pone.0309406)
Supplement: S2 Appendix — (DOCX) [file pone.0309406.s002.docx]

**S2 Appendix. Summary of the results of regression models not presented in the main part of the paper.**

Table S3 and S4 present a summary of the regression models relating fascicle length, muscle volume, physiological cross-sectional area, and anatomical cross-sectional area for the lateral and medial gastrocnemius to Achilles tendon moment arm (ATMA) assessed at 5°, 10°, and 15° of plantarflexion. Similar correlations between gastrocnemius architecture variables and ATMA in neutral ankle position are presented in the main text of the paper. Overall, the correlations were consistent as the angle at which ATMA was measured was varied. In some instances, a significant correlation is not found for one set of ATMA values, but in such cases the p-value is approaching the level of significance.

**Table S3. Correlation coefficients and p-values for simple regressions between lateral gastrocnemius fascicle length, muscle volume, physiological cross-sectional area (PCSA), and anatomical cross-sectional area (ACSA) and Achilles tendon moment arm (ATMA) assessed at 5° plantarflexion (PF), 10° plantarflexion, and 15° plantarflexion.**

|  | | **ATMA at 5° PF (mm)** | | | **ATMA at 10° PF (mm)** | | | **ATMA at 15° PF (mm)** | |
| --- | --- | --- | --- | --- | --- | --- | --- | --- | --- |
| **outcome variable** | **r** | | **p** | **r** | | **p** | **r** | | **p** |
| **fascicle length (mm)** | 0.530 | | 0.035* | 0.530 | | 0.035* | 0.452 | | 0.091 |
| **muscle volume (**$\mathbf{c}\mathbf{m}^{\mathbf{3}}$**)** | 0.610 | | 0.012* | 0.618 | | 0.011* | 0.623 | | 0.012* |
| **PCSA (**$\mathbf{c}\mathbf{m}^{\boldsymbol{2}}$**)** | 0.304 | | 0.252 | 0.308 | | 0.246 | 0.363 | | 0.183 |
| **ACSA (**$\mathbf{c}\mathbf{m}^{\boldsymbol{2}}$**)** | 0.515 | | 0.041* | 0.523 | | 0.038* | 0.553 | | 0.033* |

Asterisk (*) denotes statistically significant correlations at the α = 0.05 level.

**Table S4. Correlation coefficients and p-values for simple regressions between medial gastrocnemius fascicle length, muscle volume, physiological cross-sectional area (PCSA), and anatomical cross-sectional area (ACSA) and Achilles tendon moment arm (ATMA) assessed at 5° PF, 10° PF, and 15° PF ankle angles.**

|  | | **ATMA at 5° PF (mm)** | | | **ATMA at 10° PF (mm)** | | | **ATMA at 15° PF (mm)** | |
| --- | --- | --- | --- | --- | --- | --- | --- | --- | --- |
| **outcome variable** | **r** | | **p** | **r** | | **p** | **r** | | **p** |
| **fascicle length (mm)** | 0.276 | | 0.301 | 0.284 | | 0.286 | 0.184 | | 0.511 |
| **muscle volume (**$\mathbf{c}\mathbf{m}^{\mathbf{3}}$**)** | 0.618 | | 0.011* | 0.619 | | 0.011* | 0.607 | | 0.017* |
| **PCSA (**$\mathbf{c}\mathbf{m}^{\boldsymbol{2}}$**)** | 0.489 | | 0.055 | 0.480 | | 0.060 | 0.516 | | 0.049* |
| **ACSA (**$\mathbf{c}\mathbf{m}^{\boldsymbol{2}}$**)** | 0.523 | | 0.038* | 0.508 | | 0.045* | 0.518 | | 0.048* |

Asterisk (*) denotes statistically significant correlations at the α = 0.05 level.
