# Supplementary material for: Correlations between Achilles tendon moment arm and plantarflexor muscle architecture variables
Source: PLoS One. 2024 Aug 29;19(8):e0309406. doi: 10.1371/journal.pone.0309406 (PMC11361577; doi:10.1371/journal.pone.0309406)
Supplement: Attachment — Submitted filename: Review.pdf [file pone.0309406.s003.pdf]

Review: Correlations between Achilles Tendon Moment Arm and Plantarflexor Muscle Architecture Variables

This study aimed to investigate the *in vivo* relationship between medial and lateral gastrocnemius size and length and the Achilles tendon moment arm (ATMA). They found significant positive correlations between fascicle length and ATMA for the lateral gastrocnemius, but not for the medial gastrocnemius. In contrast, all muscle size parameters were correlated with ATMA. Overall, they conclude that the lateral gastrocnemius adapts to moment arms to maintain force production throughout the range of motion across individuals, while the medial gastrocnemius does not and is thus better suited for static force generation.

Generally, the manuscript is excellent and I thoroughly enjoyed reading it. I only have a very few minor comments and questions. Please see these below.

Line 123 - There is an error in your stature units – this should be m not cm according to your values.

Volume measures – out of interest, why did you take volume measures lying down at rest, but all other measures when standing? Would this have affected your correlations at all?

ATMA - At what point/location of the Achilles tendon were measurements obtained? Over the free tendon? Was/how was this standardised between participants?

Figures – I assume this is the online image compressor, but figure quality appears poor, so may be worth checking. E.g., in figure 1 a and b, it is difficult to see the contract of US images.
